# 10 Hz tACS Over Somatosensory Cortex Does Not Modulate Supra-Threshold Tactile Temporal Discrimination in Humans

**DOI:** 10.3389/fnins.2019.00311

**Published:** 2019-04-03

**Authors:** Marc A. Wittenberg, Mitjan Morr, Alfons Schnitzler, Joachim Lange

**Affiliations:** ^1^Institute of Clinical Neuroscience and Medical Psychology, Medical Faculty, Heinrich-Heine-Universität Düsseldorf, Düsseldorf, Germany; ^2^Division of Medical Psychology, University of Bonn, Bonn, Germany

**Keywords:** transcranial alternate current stimulation, tactile discrimination, alpha oscillations, somatosensory, supra-threshold

## Abstract

Perception of physical identical stimuli can differ over time depending on the brain state. One marker of this brain state can be neuronal oscillations in the alpha band (8–12 Hz). A previous study showed that the power of prestimulus alpha oscillations in the contralateral somatosensory area negatively correlate with the ability to temporally discriminate between two subsequent tactile suprathreshold stimuli. That is, with high alpha power subjects were impaired in discriminating two stimuli and more frequently reported to perceive only one stimulus. While this previous study found correlative evidence for a role of alpha oscillations on tactile temporal discrimination, here, we aimed to study the causal influence of alpha power on tactile temporal discrimination by using transcranial alternating current stimulation (tACS). We hypothesized that tACS in the alpha frequency should entrain alpha oscillations and thus modulate alpha power. This modulated alpha power should alter temporal discrimination ability compared to a control frequency or sham. To this end, 17 subjects received one or two electrical stimuli to their left index finger with different stimulus onset asynchronies (SOAs). They reported whether they perceived one or two stimuli. Subjects performed the paradigm before (pre), during (peri), and 25 min after tACS (post). tACS was applied to the contralateral somatosensory-parietal area with either 10, 5 Hz or sham on three different days. We found no significant difference in discrimination abilities between 10 Hz tACS and the control conditions, independent of SOAs. In addition to choosing all SOAs as the independent variable, we chose individually different SOAs, for which we expected the strongest effects of tACS. Again, we found no significant effects of 10 Hz tACS on temporal discrimination abilities. We discuss potential reasons for the inability to modulate tactile temporal discrimination abilities with tACS.

## Introduction

Perception does not only depend on the incoming stimuli, but also on intrinsic neuronal activity (or so called brain states). This intrinsic neuronal activity fluctuates over time and from trial to trial. Recent studies have shown that such fluctuations of neuronal activity can substantially influence perception. Specifically, fluctuations of neuronal oscillatory activity in the alpha band (∼8–12 Hz) correlate with perception of physical identical stimuli over time. For example, the ability to detect visual near-threshold stimuli improved with lower posterior prestimulus alpha band power (Hanslmayr et al., 2007; van Dijk et al., 2008). Similarly in the somatosensory domain, lower prestimulus alpha band power was related to better perception or discrimination of tactile stimuli (Linkenkaer-Hansen et al., 2004; Haegens et al., 2011; Lange et al., 2012; Baumgarten et al., 2016). Alpha oscillations are therefore interpreted as reflecting the excitability of a brain area, a decision bias or active inhibition of brain areas (Thut et al., 2006; Klimesch et al., 2007; Jensen and Mazaheri, 2010; Lange et al., 2013, 2014; Iemi et al., 2017; Limbach and Corballis, 2017). The evidence for a role of prestimulus alpha power, however, is mostly correlative. To provide causal evidence for an influence of alpha power on perception it is required to modulate alpha power and measure its impact on perception.

One potential method to modulate neuronal oscillations is transcranial alternating current stimulation (tACS). tACS is a method to non-invasively stimulate the brain with electrical activity of a given frequency (Antal and Paulus, 2013). It has been suggested that tACS with 10 Hz entrains the endogenous alpha band power in the stimulated brain area during stimulation (Helfrich et al., 2014b; Ruhnau et al., 2016). Alterations in alpha power have also been shown to outlast tACS, such that alpha power was increased after tACS (Zaehle et al., 2010; Neuling et al., 2013; Kasten et al., 2016). However, these studies were not conducted in the somatosensory domain. Recently, a study in the somatosensory cortex showed a decrease in alpha power after tACS (Gundlach et al., 2017). This opens the possibility to study the causal influence of alpha oscillations on brain functions. tACS over the sensory area areas has been used successfully to elicit sensations in the respective sensory domains (Abd Hamid et al., 2015). For example, Feurra et al. (2011b) used tACS to stimulate the primary somatosensory cortex and could elicit tactile sensations in the contralateral hand. Also, tACS has been successfully used to modulate performance in motor (Pogosyan et al., 2009; Feurra et al., 2011a; Joundi et al., 2012), perceptual (Laczó et al., 2012; Neuling et al., 2012; Helfrich et al., 2014a; Kar and Krekelberg, 2014), and higher cognitive function tasks (Santarnecchi et al., 2013).

Here, we aimed to use tACS to study a putative causal impact of alpha oscillations on tactile temporal perception. A recent study has shown that prestimulus alpha band (∼8–12 Hz) power significantly negatively correlated with subjects’ ability to perceive two electro-tactile stimuli as two separate stimuli (rather than one single stimulus; Baumgarten et al., 2016). To this end, we stimulated the somatosensory cortex with tACS at 10 Hz (i.e., in the alpha band) while subjects performed a tactile temporal discrimination task (Baumgarten et al., 2016). We hypothesized that 10 Hz tACS entrains intrinsic alpha oscillations and thus modulates the power of these alpha oscillations. Subsequently, discrimination of two subsequent tactile supra-threshold stimuli is expected to be altered with 10 Hz tACS compared to sham stimulation and stimulation with a control frequency (5 Hz). We tested this hypothesis during stimulation and 25 min after stimulation had ended.

## Materials and Methods

### Subjects

We measured 17 subjects (nine female; age: 25.4 ± 1.4 years; mean ± SEM; range: 18 to 41 years). All subjects were right-handed according to the Edinburgh Handedness Inventory (87.0 ± 3.4; mean ± SEM; Oldfield, 1971).

Exclusion criteria were history or family history of epilepsy, history of loss of consciousness, brain related injury, or other neurological or psychiatric disorders, high blood pressure, cardiac pacemaker or intracranial metal implantation, tinnitus, intake of central nervous system-affective medication, pregnancy, and impairments of the peripheral nerves in the left arm.

The experiment was conducted in accordance with the Declaration of Helsinki and approved by the local ethics committee of the Heinrich-Heine-Universität Düsseldorf, Germany (Study No. 4965R). Prior to the experiment, subjects gave written informed consent.

Subjects were naïve with respect to the hypotheses and stimulation conditions. Subjects received 50€ after completion of the entire experiment.

### Paradigm

The paradigm was modified after Baumgarten et al. (2016). Subjects received one or two electrical stimuli with different stimulus onset asynchronies (SOAs) on their left index finger. Subjects were asked to respond whether they perceived one or two stimuli.

**FIGURE 1:**
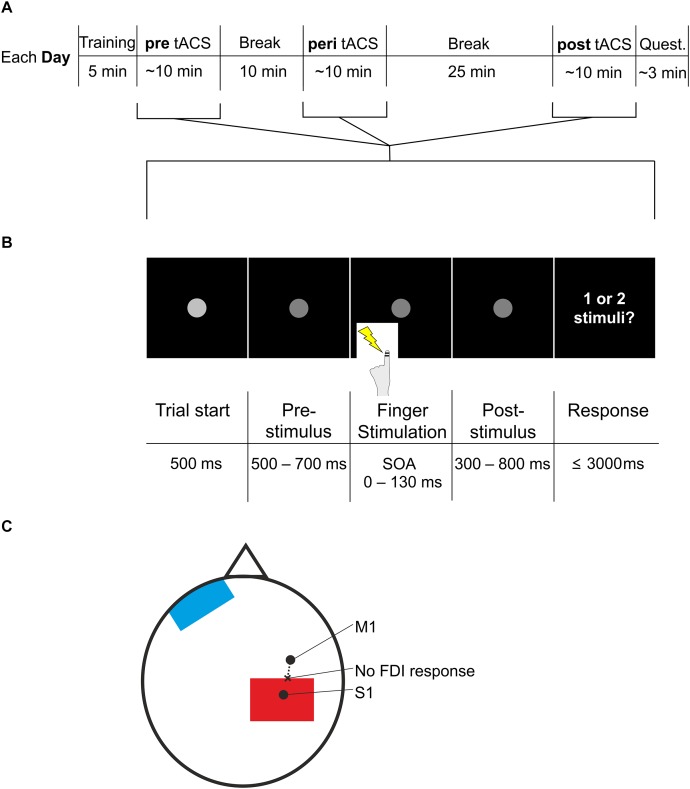
Experimental procedure and paradigm. **(A)** The experiment started with a short training period. Next, participants conducted the task (*pre*, see **B**), followed by a 10 min break. Next, participants conducted the task again, now with additional tACS (*peri*), followed by a 25 min break. Finally, participants conducted the task for the third time, now again without tACS (*post*), followed by the questionnaire. Participants repeated the entire procedure on three different days. Each day differed only in stimulation frequency of tACS (10, 5 Hz, or sham) during the *peri* section. Quest., questionnaire. **(B)** The task used in the pre, peri, and post session (see **A**) started with a fixation point, which decreased in luminance after 500 ms. This darker fixation point was shown for a jittered period of 500–700 ms. The jittered period was followed by electric stimulation of the left index finger with varying SOAs (0, 20, 30, 40, 50, 60, 70, 80, 90, 100, 110, 130 ms). After another jittered period of 300–800 ms showing the fixation point, participants were asked to respond whether they perceived one stimulus or two stimuli. Then the next trial started with the brighter fixation point. **(C)** Electrode placement. S1 was determined by neuronavigation. M1 was determined by the strongest FDI response when TMS was applied. Starting from M1, we applied TMS in steps of 0.5 cm moving to posterior (dashed line), until FDI response was no longer visible (“no FDI response”). At this spot we placed the most anterior border of the stimulation electrode (red). The reference electrode (blue) was placed on the contralateral forehead.

Each trial began with a fixation dot which decreased in luminance after 500 ms, indicating the upcoming application of the stimuli (Figure 1B). After a jittered period of 500–700 ms, subjects received one or two stimulations to the left index finger (stimulation duration: 0.3 ms each) while viewing the fixation dot. Amplitude of the stimuli was individually determined such that subjects could clearly perceive the stimuli without being painful (2.1 ± 0.2 mA; mean ± SEM). After another jittered period of 300–800 ms showing the fixation dot, subjects were asked by written instruction on the screen to respond with their right hand by button press. In nine subjects, button press with the right index finger related to perception of two stimuli and button press with the right middle finger related to perception of one stimulus. In the other subjects, button press pattern was reversed such that a press with the right index finger related to perception of one stimulus and button press with the right middle finger related to perception to two stimuli.

We used the following SOAs: 0 (i.e., only one stimulus applied), 20, 30, 40, 50, 60, 70, 80, 90, 100, 110, 130 ms. Trials with SOAs 0, 110, and 130 ms were each presented in 10 trials whereas each of the other SOAs was presented in 20 trials. SOAs with only 10 trials were added so that subjects responded to SOAs that clearly allowed for a perception of either 1 or 2 stimuli. The lower number of stimuli was chosen to keep the duration of the experiment within the time limit for tACS safety conditions (see below). The different SOAs were presented in pseudo-random order.

Subjects were asked to perform the experiment on 3 days, each separated by 1 week. On each day a different tACS frequency was applied: 10, 5 Hz, or sham. The order of tACS frequencies was randomized across subjects and double-blinded. For the double blinding, a person naïve to the experiment randomly selected the tACS frequency in each session and operated the DC stimulator during the experiment while the participants and the main experimenter who performed and analyzed the tACS experiment and communicated with the participants were unaware of the tACS frequency. Main experimenter and participants learned of the used tACS frequencies only after all three frequencies had been applied.

During each day, subjects performed the paradigm three times: pre (before tACS), peri (during tACS), and post (after tACS). The peri session started 10 min after pre session ended; the post-session started 25 min after the peri session ended (Figure 1A). The pre session was included as baseline performance of the paradigm. The post-session was included because it was shown that tACS effects can outlast the end of stimulation (Veniero et al., 2015). There is no consistent pattern, however, regarding the latency and duration of post-stimulation tACS effects (Veniero et al., 2015). While some studies report aftereffects a few minutes after the end of stimulation (e.g., Helfrich et al., 2014b), other studies report that aftereffects of 10 Hz tACS can last for 30 min (Neuling et al., 2013) or even start only 30 min after stimulation (Wach et al., 2013; see Veniero et al., 2015 for an overview). Most of these studies investigated tACS in the visual domain. Here, we aimed to investigate whether post-stimulation effects might be obtained in the somatosensory domain. Previous studies in the sensorimotor domain reported no effects of 10 Hz tACS directly after stimulation (Wach et al., 2013; Gundlach et al., 2016) and that aftereffects were visible only 30 min after stimulation (Wach et al., 2013). Therefore, we chose to study potential post-stimulation effects 25 min after tACS.

One session including all SOAs and repetitions lasted ∼8–10 min.

A training phase of 5 min was included at the beginning of each day to let subjects familiarize with the paradigm. This training phase included SOAs 0, 20, 40, 60, 80, 100, 130, 150 ms. 0 and 150 ms appeared three times as often as the other SOAs to familiarize subjects with the clear perception of 1 or 2 stimuli, respectively.

The paradigm was presented with the Presentation software (Neurobehavioral Systems, Albany, NY, United States). Electrical stimuli at the left index finger were delivered by a stimulus current generator (DeMeTec GmbH, Langgöns, Germany).

In summary, our study included three independent variables: *frequency* (sham, 5, 10 Hz), *session* (pre, peri, post), *SOAs* (0–130 ms).

The post-session of each day was followed by a short questionnaire. In this questionnaire, subjects were interviewed if they felt a sensation during the tACS. Also, they were asked whether they thought stimulation or sham was applied and how confident they were with their answer on a scale from 1 (“very unsure”) to 10 (“very sure”). If they answered that stimulation had happened, then subjects were asked on their subjective impression of the stimulation frequency and their confidence in their judgment on a scale from 1 (“very unsure”) to 10 (“very sure”).

### Transcranial Alternating Current Stimulation (tACS)

Transcranial alternating current stimulation was applied with two saline-soaked sponge electrodes (7 cm × 5 cm) on the skin surface (DC Stimulator Plus, NeuroConn, Ilmenau, Germany). The electrodes were held in place with a rubber band covering the whole electrode. One electrode was placed over the right somatosensory cortex similar to the area found in Baumgarten et al. (2016). The other electrode was placed over the left orbit. tACS was applied at 10 or 5 Hz with a current of 1 mA (peak-to-peak amplitude, sinusoidal waveform) for a maximum of 10 min leading to a current density of 28.57 μA/cm^2^ and a total charge of 0.017 C/cm^2^. Impedance was kept below 5 kΩ. These settings are within the boundary conditions of established safety protocols for transcranial direct current stimulation (Nitsche et al., 2003). Sham stimulation consisted of only 30 s stimulation with either 10 or 5 Hz. Each stimulation session included 10 s fade-in and 10 s fade-out time. If subjects finished the paradigm before 10 min, the stimulation was terminated, resulting in an average stimulation time of 8.2 ± 0.13 min (mean ± SEM).

### Localization of Right Primary Motor and Somatosensory Cortex

Since Baumgarten et al. (2016) found a significant correlation between alpha power and tactile temporal discrimination in primary somatosensory cortex (S1) contralateral to stimulation, we aimed to stimulate contralateral (i.e., right) S1 with tACS.

To this end, the right S1 was localized by using neuronavigation (LOCALITE, Sankt Augustin, Germany) based on a standard MRI brain (MNI coordinates x = 36 mm, y = −36 mm, z = 48 mm; Bingel et al., 2004).

After locating S1 with neuronavigation, the tACS electrode can be placed differently on the located spot (i.e., electrode centered above spot or spot at the border of the electrode). We sought to place the electrode to minimally overlap with motor cortex to avoid stimulation of the finger muscle which might be misjudged for a stimulus from the finger electrodes and thus interfere with the task (Figure 1C). To this end, we localized the right primary motor cortex (M1) with TMS.

Right M1 was localized by inducing muscle twitching in the first dorsal interosseus (FDI) by means of TMS. TMS of the right motor cortex was performed using a standard figure of eight coil (MC-B70) connected to a MagPro stimulator (Medtronic, Minneapolis, MN, United States). We located the right FDI by placing the coil tangentially to the scalp with the handling pointing backward. We began by placing the coil 45° away from the head midline and vertical to the right periauricular point. Moving the coil anterior, posterior, medial, and lateral in ∼0.5 cm steps led to the localization with the maximal FDI motor response. This spot was determined as M1.

From M1 we applied TMS again posterior in ∼0.5 cm steps until hand twitching stopped. This point we determined as the posterior end of M1. Here, we placed the anterior border of the electrode.

S1 localized by neuronavigation was 2.8 ± 0.2 cm posterior to M1.

### Data Analysis and Statistics

For data analysis we used custom MATLAB (The MathWorks, Natick, MA, United States) scripts.

For each frequency (5, 10 Hz, sham), session (pre, peri, post), SOA and subject, we determined mean responses across all repetitions. Next, for each frequency, session and SOA, individual mean responses were averaged across subjects.

In our main statistical analysis, we applied three-way repeated-measures ANOVA (rmANOVA, Trujillo-Ortiz, 2006) with factors *Frequency*, *Session* and *SOAs*, after testing for normality of the data by means of Shapiro–Wilk tests (BenSaïda, 2009, all *p*-values > 0.42). The main hypothesis was to test whether subjects’ responses showed significant main effects of *Frequency* and/or *Session*, or significant interaction effects.

Since our main analysis did not reveal any relevant significant effects (see section “Results”), we performed additional statistical tests. These tests were performed to exclude the possibility that the non-significant results of the main analysis were caused by too low statistical power, by “noise” in the data due to the inclusion of data points that are irrelevant with respect to the hypothesis, or by too high intra- or inter-individual variability of responses.

The normalization was done in two different ways. In the first additional analysis, we normalized the data to minimize intra-individual variability.

The first normalization was based on the potential problem that individual performance might differ between different days in terms of absolute performance. We aimed to reduce intra-individual differences across days by normalizing the responses in the peri and post-sessions with respect to the pre session according to the formula:

(1) $$r\_{\mathrm{norm}}_{\mathrm{Freq,Session}}(\mathrm{SOA})=\frac{r_{\mathrm{Freq,Session}}(\mathrm{SOA})-r_{\mathrm{Freq,pre}}(\mathrm{SOA})}{r_{\mathrm{Freq,pre}}(\mathrm{SOA})}$$

with *r_norm* being the individual normalized mean response as a function of *SOA* for a given tACS frequency *Freq* (10, 5 Hz, Sham) and paradigm *Session* (pre, peri, post). *r* denotes the non-normalized response as a function of *SOA* for a given *Freq* and *Session.* This normalization results in a measure that can be described as “responses relative to the pre session.”

In a second normalization, we sought to reduce inter-individual differences by transforming individual mean responses on a scale between 0 and 1 according to the following formula

(2) $$r\_{\mathrm{norm}}_{\mathrm{Freq,Session}}(\mathrm{SOA})=\frac{r_{\mathrm{Freq,Session}}(\mathrm{SOA})-r\_\min_{\mathrm{Freq,Session}}}{r\_\max_{\mathrm{Freq,Session}}-r\_\min_{\mathrm{Freq,Session}}}$$

with *r_norm* being the individual normalized mean response as a function of *SOA* for a given tACS frequency *Freq* (10, 5 Hz, Sham) and paradigm *Session* (pre, peri, post). *r* denotes the non-normalized response as a function of *SOA* for a given *Freq* and *Session. r_min* and *r_max* denote the non-normalized minimum and maximum, respectively, responses across all SOAs for a given *Freq* and *Session.* As mentioned above, this normalization results in responses normalized between 0 and 1.

As for the main analysis, we applied three-way repeated-measures ANOVA (rmANOVA, Trujillo-Ortiz, 2006) with factors *Frequency*, *Session* and *SOAs* on individual and normalized mean responses, again after confirming normality by means of Shapiro–Wilk tests (BenSaïda, 2009, all *p*-values > 0.12).

In the third and final analysis, we focused on *a priori* hypotheses for chosen SOAs for the statistical analysis. The *a priori* chosen SOAs were based on results of one of our previous studies (Baumgarten et al., 2016). This MEG study found an influence of alpha power on perception for intermediate SOAs at ∼25 ms. We speculated therefore that the effect of alpha power on perception is specific for SOAs of ∼25 ms, while all other SOAs are unaffected by changes in alpha power. To this end, we selected from our study only those SOAs that are close to 25 ms. That is, we chose the responses of the SOA at 20 and 30 ms, either separately or averaged across both SOAs. For statistical analyses, we applied either planned *t*-tests or Wilcoxon sign-ranked tests, depending on whether or not input data were normally distributed (again tested by means of Shapiro–Wilk tests; BenSaïda, 2009).

Alternatively, the effect of alpha power on response rates might not be specific for SOAs of 25 ms *per se*, but rather for individual intermediate SOAs (intermediate SOAs and SOAs of ∼25 ms coincide in Baumgarten et al., 2016). In the present study, the intermediate SOA was 54.1 ± 7.7 ms (mean ± SEM). If the influence of alpha power is specific for intermediate SOAs, we might expect an influence at ∼54 ms (the intermediate SOA). In this analysis, we therefore chose to analyze the effect of tACS on mean responses for the individual intermediate SOA.

In line with the statistical analyses above, we applied either planned *t*-tests or Wilcoxon sign-ranked tests, depending on whether or not input data were normally distributed (again tested by means of Shapiro–Wilk tests; BenSaïda, 2009).

For the statistical analysis of specific SOAs, we applied left-tailed tests when comparing mean responses at peri 10 Hz tACS against mean responses pre 10 Hz tACS, peri 5 Hz tACS, or peri sham tACS, respectively.

We used two-tailed tests when comparing mean responses at post 10 Hz tACS against mean responses pre 10 Hz tACS, post 5 Hz tACS, or post-sham tACS, respectively.

In addition, we used Bayesian statistics to test whether our data is in favor of the null hypothesis that there is no difference between 10 Hz tACS and control conditions. For all Bayesian tests we used the program JASP (JASP Team, 2018).

For non-normalized and normalized data, we calculated Bayesian repeated measures ANOVAs with factors *Frequency*, *Session*, and *SOAs*. For the interactions *Frequency* × *Session*, *Frequency* × *SOAs*, *Session* × *SOAs* and *Frequency* × *Session* × *SOAs* we calculated the Bayes Inclusion Factor (BF_Inclusion_) based on matched models in JASP.

For our hypotheses for specific SOAs, we calculated Bayesian paired sample *t*-tests. As with our frequentist approach, we calculated left-tailed tests for peri tACS at 10 Hz vs. control conditions (i.e., mean responses at 10 Hz tACS smaller than mean responses at control conditions), and two-tailed tests for post-tACS at 10 Hz vs. control conditions. All Bayesian statistics were estimated based on a uniform prior distribution.

As an additional analysis we tested whether subjects that reported a flicker during tACS at 10 Hz showed a behavioral effect. To this end, we compared mean responses for peri tACS at 10 Hz vs. peri tACS at sham in line with above described analyses, but now only for subjects that reported a flicker sensation.

Given that tACS can have after-effects due to neuro-plastic changes (Veniero et al., 2015), we compared the first and the second half of the trials for peri tACS at 10 Hz by means of two-way repeated measures ANOVAs for non-normalized and normalized data with factors *SOAs* and *Half* (i.e., first or second half of the trials). Beforehand, we tested data for normality by means of Shapiro–Wilk tests. All data were normally distributed (all *p* > 0.10). Additionally, we calculated Bayesian repeated measures ANOVAs with factors *SOAs* and *Half*.

We also tested the first half against the second half of the trials for peri tACS at 10 Hz for the aforementioned specific SOAs. Depending on normality (tested by Shapiro–Wilk tests) we applied either planned *t*-tests or planned Wilcoxon sign-ranked tests. Additionally, we calculated Bayesian *t*-tests.

## Results

### Questionnaire

All subjects tolerated tACS and TMS well. Four subjects felt a tingling sensation under the electrodes at the start of the stimulation. Four subjects reported a light burning under an electrode at the beginning of the stimulation while one of them felt the burning during the whole stimulation at 10 Hz. Two subjects reported a warming under an electrode.

Five subjects had a flickering effect in their visual field at 10 Hz tACS. Two subjects had the flickering only at the beginning of the stimulation while three subjects during the whole stimulation.

When 10 Hz tACS was applied, two of the 17 subjects correctly identified the 10 Hz frequency with a confidence rating of 7.0 ± 0.3 (mean ± SEM), only one of them reporting the flickering effect.

For the 5 Hz tACS frequency, five of the 17 subjects identified correctly the 5 Hz frequency with a confidence rating of 3.2 ± 0.9. For sham tACS, six of the 17 subjects identified correctly that sham tACS was applied with a confidence rating of 5.8 ± 0.6. Since all these values are below chance level, we evaluated the blinding procedure as successful.

### General Effects of 10 Hz tACS on Tactile Perception

**FIGURE 2:**
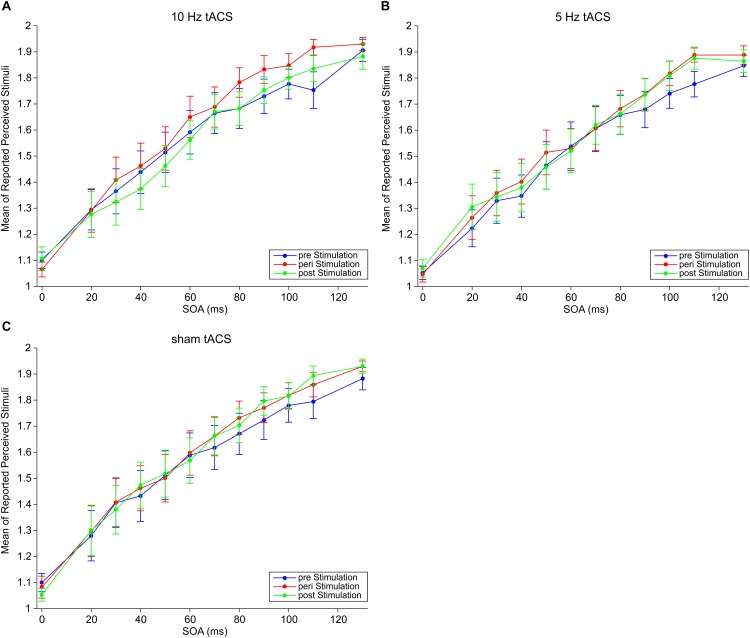
Mean responses of perceived stimuli at different SOAs for **(A)** 10 Hz tACS, **(B)** 5 Hz tACS, and **(C)** sham tACS before (pre), during (peri), and 25 min after (post) stimulation. Error bars represent SEM.

We measured perceptual responses in a temporal tactile discrimination task where subjects had to decide whether they perceived one or two electrical stimuli. We employed tACS at three different stimulation conditions: 10, 5 Hz, and sham. For each tACS frequency, subjects performed the paradigm three times: pre-, peri-, and post-tACS. Mean responses are shown in Figure 2. We tested the hypothesis that tACS at 10 Hz should modulate subjects’ perception.

Three-way repeated measures ANOVA (rmANOVA) with factors *Frequency* (sham, 5, 10 Hz), *Session* (pre, peri, post), and *SOAs* (0–130 ms) revealed no significant main effects of *Frequency* [*F*(2,32) = 0.78, *p* = 0.47], *Session* [*F*(2,32) = 1.67, *p* = 0.20], nor interaction effects for *Frequency* × *Session F*(4,64) = 0.64, *p* = 0.64], *Frequency* × *SOAs* [*F*(22,352) = 0.44, *p* = 0.99], and *Frequency* × *Session* × *SOAs* [*F*(44,704) = 0.72, *p* = 0.91]. There was a significant main effect of *SOAs* [*F*(11,176) = 59.59, *p* < 0.01] which indicates that mean responses increase with increasing SOAs (Figure 2). There was also a significant interaction *Session* × *SOAs* [*F*(22,352) = 2.29, *p* < 0.01] which indicates that the increase of mean responses over SOAs differs between sessions independent of tACS frequency. However, the aim of our study was to investigate an effect of tACS frequency. Therefore, these two significant effects are irrelevant with respect to the main goal and will thus not further be discussed.

Bayesian repeated measures ANOVA with factors *Frequency*, *Session*, and *SOAs* revealed Bayes factors in favor of the null hypothesis that there is no difference in mean responses for the relevant main factors *Frequency* and *Session* and the interactions (*Frequency*: BF_10_ = 0.11, *Session*: BF_10_ = 0.07, *Frequency* × *Session*: BF_Inclusion_ = 0.01, *Frequency* × *SOAs*: BF_Inclusion_ = 6.37 × 10^−6^, *Session* × *SOAs*: BF_Inclusion_ = 3.93 × 10^−5^, *Frequency* × *Session* × SOAs: BF_Inclusion_ = 8.89 × 10^−6^). Only the factor *SOAs* revealed strong evidence for the alternative hypothesis (BF_10_ = 6.50 × 10^346^)^,^ indicating that the factor SOA is an explanatory factor for the observed pattern of the data. Since this factor is of no relevance for the hypothesis of our study, we will not further discuss this finding.

Since the most relevant effects in the above analyses were not significant, we conducted further analyses to exclude several factors that might have hampered the main analyses. Our approaches included normalization approaches (to reduce intra- and inter-subjective variability) or using specific *a priori* hypotheses based on previous results (Baumgarten et al., 2016; see section “Materials and Methods”).

### Normalized Response Rates

We normalized data in two ways: in a first approach, we normalized individual mean responses relative to the pre session for each tACS frequency. In the second approach, we normalized individual mean responses relative to individual minimum and maximum mean responses.

Similar to the main analysis of non-normalized response rates, we only obtained significant results for the main factor *SOAs* [relative to pre: *F*(11,176) = 2.83, *p* < 0.01; relative to minimum-maximum: *F*(11,176) = 61.56, *p* < 0.01] and the interaction factor *Session* × *SOA* [relative to pre: *F*(22,352) = 2.14, *p* < 0.01; relative to minimum-maximum: *F*(22,352) = 1.67, *p* = 0.03]. Again, because these results are not relevant for our main goal, no *post hoc* analyses were carried out here.

We did not obtain significant results for main factors *Frequency* and *Session* nor for the interactions *Frequency* × *Session*, *Frequency* × *SOAs*, or *Frequency* × *Session* × *SOAs* (relative to pre: all *p* > 0.08; relative to minimum-maximum: all *p* > 0.15).

When data were normalized to the pre session, we obtained large Bayes factors for *Session* (BF_10_ = 29913.82) and *SOAs* (BF_10_ = 3.80). The large Bayes factor for the main factor Session most likely indicates a trivial result. Due to the normalization, all values in the pre session are set to “0” whereas the values in the peri and post-session are non-zeros. Bayesian analysis states that the model “Session” explains this difference better than a randomized model between all values. However, in this case this does not reveal a true difference between sessions *per se* but rather this is a result of our normalization procedure.

The main factor *Frequency* provides evidence for no difference between tACS frequencies (BF_10_ = 0.02). Also, the Bayes factors for the interactions provided strong evidence in favor of no effects (*Frequency* × *Session*: BF_Inclusion_ = 0.06, *Frequency* × *SOAs*: BF_Inclusion_ = 4.83 × 10^−5^, *Session* × *SOAs*: BF_Inclusion_ = 9.00 × 10^−4^, *Frequency* × *Session* × *SOAs*: BF_Inclusion_ = 2.82 × 10^−5^).

When data was normalized relative to minimum-maximum, Bayesian repeated measures ANOVA revealed again Bayes factors in favor of the null hypothesis that there is no difference in mean responses for the relevant factors (*Frequency*: BF_10_ = 0.02, *Session*: BF_10_ = 0.02, *Frequency* × *Session*: BF_Inclusion_ < 0.01, *Frequency* × *SOAs*: BF_Inclusion_ = 1.75 × 10^−5^, *Session* × *SOAs*: BF_Inclusion_ = 1.77 × 10^−5^, *Frequency* × *Session* × *SOAs*: BF_Inclusion_ = 9.82 × 10^−6^). Only the factor *SOAs* provided strong evidence for an effect (*SOAs*: BF_10_ = 1.31 × 10^399^), indicating again that the factor *SOAs* is an explanatory factor for the observed pattern of the data. Since this factor is of no relevance for the hypothesis of our study, we will not further discuss this finding.

### Comparison Between the First and Second Half of the Trials for 10 Hz tACS

To test whether tACS duration influences perception (e.g., due to neuro-plastic changes), we compared the first and the second half of the trials for the peri session of tACS at 10 Hz.

A two-way repeated measures ANOVA revealed neither a significant main effect for *Half* nor an interaction effect for *SOAs* × *Half* (all *p* > 0.22 for normalized and non-normalized data and for *a priori* chosen SOAs).

Bayesian statistics provided evidence for no difference between halves (all BF_10_ < 0.20, for normalized and non-normalized data). Results for the interaction *SOAs* × *Half* provided evidence for no interaction effects (all BF_Inclusion_ ≤ 0.23).

### *A priori* Hypotheses for the Effect of 10 Hz tACS on Tactile Perception at Intermediate SOAs

Here, we test the hypothesis that 10 Hz tACS might affect specifically intermediate SOA (i.e., SOAs for which subjects had mean responses of ∼1.5, i.e., no clear bias toward perception of “1” or “2”).

**FIGURE 3:**
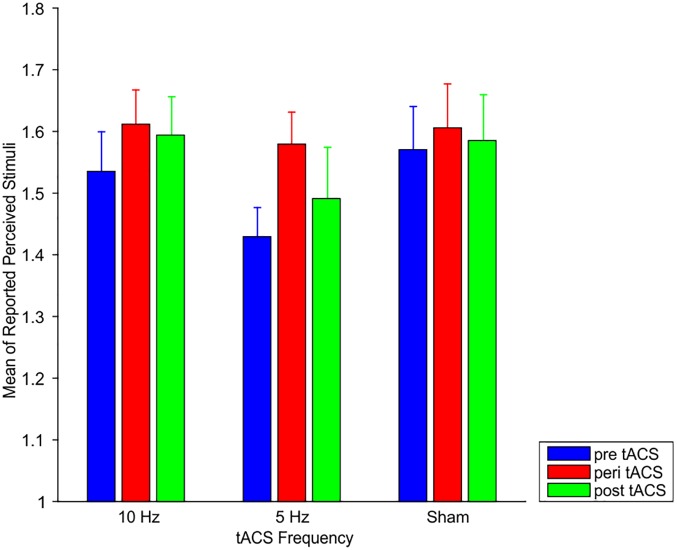
Mean responses of perceived stimuli at the individual intermediate SOA for 10 Hz tACS, 5 Hz tACS, and sham tACS before (pre), during (peri), and 25 min after (post) stimulation. Error bars represent SEM.

Mean responses at peri 10 Hz tACS did not differ significantly from mean responses at pre 10 Hz tACS, peri Sham tACS or peri 5 Hz tACS (all *p* > 0.54; Figure 3). Bayesian statistics provided evidence for the null hypothesis of no effect of tACS (all BF_10_ < 0.23).

Likewise, mean responses at post 10 Hz tACS did not differ significantly from mean responses at pre 10 Hz tACS, post-Sham tACS or post 10 Hz tACS (all *p* > 0.34; Figure 3). Bayesian statistics provided either inconclusive results or evidence for the null hypothesis of no effect of tACS (all BF_10_ between 0.25 and 0.44).

### Hypotheses for the Effect of 10 Hz tACS on Tactile Perception at SOAs 20 and 30 ms

A previous study reported a correlation of alpha power and perception at SOAs of ∼25 ms (Baumgarten et al., 2016). Therefore, we tested in this analysis that the causal effect of 10 Hz oscillations on temporal tactile perception might not be related to the intermediate SOA *per se*, but rather to an SOA of 20 to 30 ms.

Mean responses at peri 10 Hz tACS did not differ significantly from pre 10 Hz tACS, peri Sham tACS or peri 5 Hz tACS at an SOA of 20, 30 ms, or when responses of the SOAs at 20 and 30 ms where combined (all *p* > 0.38). Bayesian statistics provided evidence in favor of the null hypothesis (all BF_10_ < 0.27).

Likewise, mean responses at post 10 Hz tACS did not differ significantly from mean responses at pre 10 Hz tACS, post-Sham tACS or post 10 Hz tACS (all *p* > 0.22). Bayesian statistics provided either inconclusive results or evidence for the null hypothesis of no effect of tACS (all BF_10_ between 0.26 and 0.48).

### Additional Analyses Only for Subjects That Reported a Flicker Sensation

When comparing mean responses for peri tACS at 10 Hz vs. peri tACS at sham only for subjects that reported a flicker sensation, there was no behavioral effect (all *p* > 0.21).

## Discussion

We stimulated the somatosensory cortex with transcranial tACS while subjects performed a tactile discrimination task. Based on previous findings that reported a correlation between alpha power and tactile discrimination abilities (Baumgarten et al., 2016), we hypothesized that 10 Hz tACS would affect subjects’ tactile perception. This way, we would provide evidence for a causal role of alpha power for tactile perception and add on the numerous studies reporting a correlation between (prestimulus) alpha power and perception. However, we found no significant effects of 10 Hz tACS on perceptual performance, neither when applied while subjects performed the task (i.e., peri tACS) nor did we find any aftereffects of stimulation (post-tACS).

That is, we did not find evidence for a causal role of alpha oscillations for tactile temporal discrimination. Bayesian statistics revealed that there is moderate to strong evidence in favor of the null hypothesis that mean responses with tACS at 10 Hz do not differ from control conditions. That is, our results are in favor that tACS at 10 Hz did not modulate tactile temporal discrimination. However, we do not conclude that 10 Hz or alpha power is not causally involved in tactile temporal discrimination. For such a conclusion there are still many factors to be considered as discussed below.

We will discuss in the following potential reasons and implications of this null result.

One potential reason might be that tACS at 10 Hz did not entrain neuronal oscillations. Since we did not measure neuronal activity in our study, we cannot exclude this possibility. Several previous studies, however, have shown that tACS in the alpha-band modulates neuronal oscillations. These studies have shown that alpha power is typically increased during tACS (Helfrich et al., 2014b; Ruhnau et al., 2016) as well as after tACS (Zaehle et al., 2010; Neuling et al., 2013; Kasten et al., 2016). In contrast to our study, these studies were not conducted in the somatosensory domain. In the somatosensory domain, recently, a decrease of alpha power after tACS at alpha frequencies was reported (Gundlach et al., 2017). One might argue that the current density we used may have been too low to entrain neuronal oscillations. Several studies, however, were able to entrain brain oscillations using similar current densities as we did (Moliadze et al., 2012; Neuling et al., 2015; Ruhnau et al., 2016). Since these studies were conducted in the visual domain, it might still be that in the somatosensory domain stronger current densities are needed to induce behavioral relevant entrainment. However, we refrained from using higher current densities because Feurra et al. (2011b) showed that tACS with a higher current density over S1 at alpha frequency elicited tactile sensations in the contralateral hand. Therefore, we used lower current density to minimize the possibility of inducing tactile sensations interfering with the task.

Another potential problem might be spatial inaccuracies in the stimulation so that our tACS did not entrain neuronal oscillations in S1. To exclude such a problem, we located S1 with two independent criteria (neuronavigation and no motor response with TMS) and we applied a large stimulation electrode. It seems thus unlikely that a putative entrainment did not affect S1.

In sum, although we have no direct measure of entrainment, we are confident that we entrained neuronal oscillations in the same area in which alpha power correlated with tactile discrimination in our previous study (Baumgarten et al., 2016).

Previous studies reported no unequivocal effects of tACS on perception. On the one hand, studies reported that tACS modulates perception (Neuling et al., 2012; Brignani et al., 2013; Gundlach et al., 2016; Veniero et al., 2017). On the other hand, several studies did not find an effect of tACS on perception (Brignani et al., 2013; Gundlach et al., 2016; Veniero et al., 2017; Sheldon and Mathewson, 2018). Specifically in the somatosensory domain, results are not clear. For example, Sliva et al. (2018) reported that tACS at alpha frequencies over somatosensory cortex lead to a decrease of performance in a tactile detection task of near-threshold stimuli. This decrease was reported for baseline corrected detection rates, but not for absolute detection rates. Thus, the putative effect of tACS may at least partially be explained by differences in baseline performances. In contrast, Gundlach et al. (2016) reported for a similar task that tACS at alpha frequencies did not affect mean detection rates. However, they reported that detection rates varied in a phasic manner, i.e., depending on the phase of tACS. Notably, these studies used detection tasks in which subjects had to report whether a stimulus near perceptual threshold was perceived. In our study, however, we used a discrimination task in which stimulation was always above perceptual threshold and subjects had to report whether they perceived one or two stimuli. Detection and discrimination tasks might be influenced by different processes. For example, our previous studies have shown that tactile discrimination tasks are influenced by power in the alpha frequencies, but the phase of beta frequencies (Baumgarten et al., 2015, 2016). Therefore, we focused our analysis on power modulations. In line with this hypothesis, Brignani et al. (2013) reported an effect of tACS at alpha frequencies in a visual detection task, while they could not find an effect of 10 Hz tACS in a visual discrimination task. Future studies might explore the differences between detection and discrimination tasks and how tACS might affect these tasks in more detail.

There is no clear consensus which frequency to use when tACS with “alpha frequencies” is applied. Whereas some studies used individual alpha frequencies, based on individual peak frequencies of neuronal oscillations in the alpha band (Cecere et al., 2015; Gundlach et al., 2016), others used a fixed frequency for all subjects (Brignani et al., 2013; Kar and Krekelberg, 2014; Sheldon and Mathewson, 2018). In the present study, we used a fixed frequency of tACS for all subjects. While this approach is easier to perform, especially since we did not measure neuronal oscillations, a fixed frequency might bear the downside that tACS does not match the “optimal” frequency in all subjects. According to the Arnold’s tongue principle, low stimulation intensities only entrain the endogenous frequency in a small frequency band, whereas higher stimulation intensities can entrain a wider frequency band around the endogenous frequency (Herrmann et al., 2016; Kurmann et al., 2018). Therefore, it could be that we did not entrain alpha power in those subjects whose endogenous peak alpha frequency differs too much from 10 Hz to be entrained at the low stimulation intensity. However, Baumgarten et al. (2017) showed that tactile temporal discrimination does not correlate with individual alpha frequency of neuronal oscillations. In addition, several studies found an effect of tACS on detection using fixed frequencies (e.g., Brignani et al., 2013; Kar and Krekelberg, 2014). Finally, Baumgarten et al. (2016) reported an effect of alpha power on discrimination performances for one frequency, averaged across all subjects, rather than individual frequencies for each subject. Therefore, it seemed feasible for us to expect an effect of a fixed frequency for tACS. On the other hand, it could be that the mechanisms underlying tactile discrimination are not modulated by 10 Hz but other, neighboring frequencies within the alpha band. Given our low stimulation intensity, this potential alpha frequency might not be entrained due to the Arnold’s tongue principle. As mentioned above, however, we were restricted to 1 mA stimulation intensities, because a higher stimulation intensity could have produced tactile sensations (Feurra et al., 2011b), which might be misjudged for a stimulus from the finger electrode and thus distort behavioral results.

One might argue that the control frequency of 5 Hz might affect alpha power similarly to 10 Hz stimulation (de Graaf et al., 2013). Given that we found no effect of tACS in our study at all, this limitation does not change the conclusion of this study.

Given that tACS can produce after-effects due to neuro-plastic changes (Veniero et al., 2015), we also investigated whether tACS at 10 Hz might have an effect only at a later time segment during the stimulation. To this end, we compared the first half of the trials to the second half of the trials during peri tACS at 10 Hz. We found no differences between the first and the second half of the trials. This result suggests that longer stimulation duration did not lead to stronger results.

In summary, in our study we were unable to modulate tactile discrimination by applying tACS at alpha frequencies contralateral to the tactile stimulation. Consequently, we were unable to provide evidence for a causal role of somatosensory alpha oscillations in tactile discrimination tasks. tACS experiments comprise many degrees of freedom (e.g., electrode placements, stimulation frequency, stimulation current density, task and combinations of all factors). Another problem is that tACS can have different effects on different individuals due to anatomical differences such as the gyral depth or the thickness of the skull (Nitsche et al., 2008; Opitz et al., 2015). These factors result in a large search space for optimal parameters for the tACS experiment, making it difficult to decide for the optimal setup with regard to the question investigated (Kar and Krekelberg, 2014). And even with identical parameters, sometimes results of an tACS experiment cannot be replicated, even within one study (Veniero et al., 2017).

We are, however, confident that we used a reasonable parameter space for the stimulation parameters to expect a modulation of discrimination abilities. Thus, we might conclude that this specific combination of experimental factors is unable to modulate tactile temporal discrimination, but that we cannot conclude whether alpha power has a causal role on tactile temporal discrimination. This null effect should thus offer new insights and increase knowledge about an adequate setup of tACS experiments and to further understand difficulties and sometimes inconsistent results in tACS studies. Nevertheless, additional studies are needed to investigate a potential causal role of somatosensory alpha oscillations in tactile discrimination tasks.

## Data Availability

The datasets generated for this study are available on request to the corresponding author.

## Author Contributions

MW and JL designed the study and analyzed the data. MW and MM collected the data. MW, MM, AS, and JL wrote the manuscript.

## Conflict of Interest Statement

The authors declare that the research was conducted in the absence of any commercial or financial relationships that could be construed as a potential conflict of interest.
